# Association between Lipid Profiles and Serum Urate: A Cross-Sectional Study in Southwestern China

**DOI:** 10.1155/2021/2741131

**Published:** 2021-07-08

**Authors:** Jiying Qi, Xiaojuan Dai, Binbin Zhou, Yang Su, Zhen Xie, Dongmei Liu

**Affiliations:** ^1^Department of Endocrine and Metabolic Diseases, Shanghai Institute of Endocrine and Metabolic Diseases, Ruijin Hospital, Shanghai Jiao Tong University School of Medicine, Shanghai, China; ^2^Shanghai National Clinical Research Center for Metabolic Diseases, Key Laboratory for Endocrine and Metabolic Diseases of the National Health Commission of the PR China, Shanghai National Center for Translational Medicine, Ruijin Hospital, Shanghai Jiao Tong University School of Medicine, Shanghai, China; ^3^Department of Rheumatology, Zhongshan Hospital, Fudan University, Shanghai, China; ^4^Department of Rheumatology, Xiamen Hospital, Zhongshan Hospital, Fudan University, Xiamen, Fujian, China; ^5^Clinical Laboratory, Sichuan Provincial People's Hospital, University of Electronic Science and Technology of China, Chengdu, China; ^6^Chinese Academy of Sciences Sichuan Translational Medicine Research Hospital, Chengdu, China; ^7^Department of Dermatology, Sichuan Provincial People's Hospital, University of Electronic Science and Technology of China, Chengdu, China

## Abstract

**Objective:**

The relationship between lipid profiles and serum urate has not been fully investigated. This study aims to investigate the sex- and age-specific association between lipid profiles and serum urate.

**Methods:**

This was a cross-sectional study involving 122,351 participants aged 18–99 years from a check-up centre in Southwestern China. Generalized additive models and smooth curve fitting were conducted to explore the association between components of lipid profiles and serum urate. Furthermore, multivariate linear and logistic regression models were also performed.

**Results:**

In generalized additive models, the fitted smoothing curves showed that serum urate fluctuated in a small range with total cholesterol, LDL-C, or HDL-C raising. After adjusting for confounders, the differences in serum urate progressively increased with raising serum triglycerides quartiles. The likelihood (odds ratio, OR) for developing serum urate > 420 *μ*mol/L significantly increased in the highest quartile of triglycerides than in the lowest quartile, in hypertriglyceridemia than in normal triglycerides, and with 1 mmol/L increment in triglycerides in all sex- and age-specific groups. Furthermore, the increased OR (95% confidence interval) was higher in females than in males compared with their respective controls.

**Conclusions:**

Serum urate and the likelihood for developing serum urate >420 *μ*mol/L increased with triglycerides raising. Females were in a higher likelihood for developing serum urate >420 *μ*mol/L than males with raising triglycerides. With changes in total cholesterol, LDL-C, or HDL-C, serum urate fluctuated in a small range.

## 1. Introduction

Uric acid is the end product of purine metabolism in humans and is mainly produced in the liver [1]. Elevated serum urate is the main risk factor for gout and is associated with a variety of chronic comorbidities, such as cardiovascular disease, chronic kidney disease, and metabolic syndrome. [2, 3]. Hyperuricemia is caused by a variety of genetic and environmental factors. Genetic risk loci, such as *SLC2A9*, *SLC22A12*, and *ABCG2*, could explain about 60% of the variance in serum urate levels [4–6]. Besides, environmental factors could also influence serum urate concentrations, such as high fructose intake, alcohol consumption, obesity, and dyslipidemia. [7–9].

The relationship between serum urate and lipid profiles has been investigated in a variety of studies [10–13]; Mendelian randomization analysis found no evidence for serum urate in causing dyslipidemia [14, 15]. However, cross-sectional studies showed that elevated serum urate was associated with higher levels of LDL-C, triglycerides, total cholesterol, and lower concentrations of HDL-C [10, 12, 13]. In a retrospective cohort study, the risk for developing high LDL-C and hypertriglyceridemia was increased with higher baseline serum urate levels [11]. In turn, the influence of lipid profiles on serum urate levels has not been fully and extensively investigated. A Mendelian randomized study found a causal role for triglycerides in increasing serum urate in males [14]. A cross-sectional study revealed a higher risk for hyperuricemia with serum triglycerides elevating [16]. However, the magnitude of the association between lipid profiles and serum urate in sex- and age-specific groups has not been fully investigated.

Thus, wwin this cross-sectional study with a large health check-up population, we studied the association between lipid profiles and serum urate and further investigated this association in sex- and age-specific groups. We aimed to fully reveal the association between lipid profiles and serum urate.

## 2. Materials and Methods

### 2.1. Subjects

This large cross-sectional study was performed using electronic records from the Health Examination Centre of Sichuan Provincial People's Hospital in southwestern China. A total of 147,404 subjects (84,736 males and 62,668 females) aged 18 to 99 years who underwent health examination were enrolled from October 2013 to April 2017. The participants came from different work places with different educational background and socioeconomic status, and they all lived in the urban area of Chengdu.

Age was determined according to each participant's birthday. Body weight was measured using digital scales in kilogram with light clothes. Body height was assessed in centimetres using electronic height measuring instrument without shoes. Body mass index (kg/m^2^) was calculated by dividing the weight in kilograms by the square of the height in metres. This study complied with the Declaration of Helsinki and was approved by the Ethics Committee of Sichuan Provincial People's Hospital. During data processing, patient identification numbers were scrambled to ensure patient privacy, and the researchers were blinded to patient identities. Patients' informed consent was exempted. The scrambled identifications of individuals were linked with their files, which included demographic data, laboratory tests, and imaging examination information.

### 2.2. Measurement of Serum Parameters

Blood samples were obtained in the morning after 10–12 hours of overnight fasting and immediately tested for serum parameters. Measurement of kidney function and lipid profiles, including serum urate, creatinine, triglycerides, total cholesterol, HDL-C, LDL-C, and fasting plasma glucose, was performed using the automatic biochemical analyzer (Olympus AU5421, Japan). The measurements were performed at the Department of Clinical Laboratory, Sichuan Provincial People's Hospital. The estimated glomerular filtration rate (eGFR, mL/min/1.73 m^2^) was calculated using the Chronic Kidney Disease-Epidemiology Collaboration formula [17].

### 2.3. Statistics

Outliers were removed from the data based on a box-and-whisker diagram. The extreme values excluded for various variables were as follows: serum urate <90 and >594 *μ*mol/L (*n* = 1,199), serum triglycerides <0.07 and >3.71 mmol/L (*n* = 9,006), total cholesterol <2.44 and >7.32 mmol/L (*n* = 1,437), HDL-C < 0.41 and >2.28 mmol/L (*n* = 1,869), LDL-C < 0.75 and >5.07 mmol/L (*n* = 702), body mass index < 14.86 and >32.65 kg/m^2^ (*n* = 1,187), fasting plasma glucose <3.44 and >6.59 mmol/L (*n* = 7,434), serum creatinine <22.3 and >113.2 *μ*mol/L (*n* = 624), and eGFR < 66.35 and >141.94 mL/min/1.73 m^2^ (*n* = 1,595). Finally, 122,351 subjects were ultimately analyzed. Of them, 55,742 were females and 66,609 were males. The quantile-quantile plot and histogram were performed to assess the normality of the distribution of the variables. Normally distributed variables were presented as mean ± SD, whereas variables with a skewed distribution were shown as median (interquartile range). The analysis of variance for continuous variables and chi-squared test for categorical variables were used to compare the characteristics of the study population among sex- and age-stratified groups.

Generalized additive models (GAMs) and smooth curve fitting were performed to explore the association between components of lipid profiles and serum urate. Based on previous literature findings, the following factors were considered as potential confounders and adjusted in GAMs, namely, age, body mass index, fasting plasma glucose, and eGFR.

The level of serum triglycerides was categorized into quartiles or normal triglycerides (<1.7 mmol/L) and hypertriglyceridemia (≥1.7 mmol/L). The statistical significance of trends was assessed using linear regression models with or without adjustment for the same confounders. Meanwhile, logistic regression models were performed using a dichotomous outcome of serum urate, namely, serum urate >420 *μ*mol/L and ≤420 *μ*mol/L, as the dependent variable. For all difference estimates and odds ratios (ORs), we calculated 95% confidence intervals (95% CIs). All statistical analyses were carried out using *R* statistical software, version 4.0.5. A *p* value < 0.05 was considered statistically significant.

## 3. Results

### 3.1. Characteristics of the Subjects

The characteristics of the subjects were shown in Table 1. For both females and males, the majority of subjects were aged 30–59 years. In females, the median age was 44 (36–52) years with a mean body mass index of 22.57 ± 3.00 kg/m^2^. The mean serum urate was 281.61 ± 58.44 *μ*mol/L with a trend of increase after 50 years. With aging, serum LDL-C, total cholesterol, triglycerides, fasting plasma glucose, and creatinine tended to increase. While eGFR gradually decreased, serum HDL-C was numerically similar in different age-stratified groups.

In males, the median age was 45 (37–54) years with a mean body mass index of 24.31 ± 2.95 kg/m^2^. With aging, serum urate had a trend of decline; serum LDL-C, total cholesterol, and triglycerides increased numerically until 50–59 years, 50–59 years, and 40–49 years, respectively; then, there was a slight decline, while serum HDL-C tended to increase after 30–39 years. Serum fasting plasm glucose progressively elevated with aging. For serum creatinine, there was a slight decrease with aging until 60–69 years, and then, there was an increase in subjects aged 70–79 years; while eGFR gradually declined (Table 1).

### 3.2. Association between Lipid Profiles and Serum Urate

**Figure 1:**
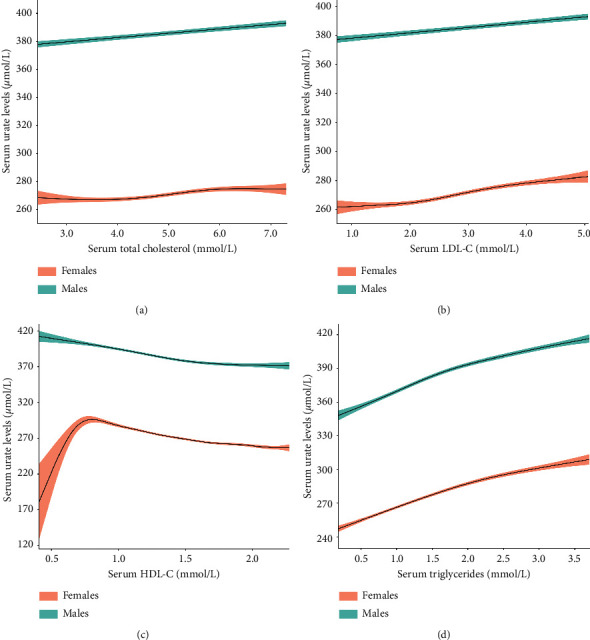
The fitted smoothing curves with 95% confidence intervals for lipid profiles and serum urate level after adjustment for age, body mass index, and estimated glomerular filtration rate. (a) Total cholesterol, (b) LDL-C, (c) HDL-C, and (d) triglycerides. LDL-C: low-density lipoprotein cholesterol; HDL-C: high-density lipoprotein cholesterol.

The fitted smoothing curves showed that serum urate increased with serum triglycerides, LDL-C, or total cholesterol raising in both females and males (Figure 1). For HDL-C, serum urate decreased as HDL-C increasing in males; while in females, serum urate increased with HDL-C elevating up to 0.81 mmol/L and thereafter decreased with further increasing HDL-C. However, it should be noted that the fitted smoothing curve was with low power between HDL-C ≥ 0.41 and < 0.81 mmol/L because only a small number of subjects were in this range (*n* = 214) (Figure 1). Since serum urate levels only fluctuated in a small range with changes in LDL-C, total cholesterol, or HDL-C, the associations between LDL-C, total cholesterol, or HDL-C and serum urate were not further analyzed.

### 3.3. Differences in Serum Urate Levels according to Serum Triglycerides Quartiles

Serum urate gradually increased with raising serum triglycerides quartiles in all participants of each sex and in each sex- and age-stratified group (Table 2). After adjusting for confounders, the increased trend remained except that in females aged <29 years in quartile 2. For females, the older the age, the higher the increment of serum urate in each quartile, compared with their respective age-specific control group (quartile 1). While for males, the increment in serum urate among different age groups had no obvious differences (Table 2).

### 3.4. The Likelihood of Having Serum Urate >420 *μ*mmol/L according to Serum Triglycerides Quartiles

In unadjusted logistic model, the likelihood for developing serum urate >420 *μ*mol/L progressively raised across serum triglycerides quartiles in both females and males (Table 3). After adjusting for confounders, the increased likelihood for developing serum urate >420 *μ*mol/L retained except females in quartile 2. In age-stratified groups, females aged 60–69 years in quartile 2, >30 years in quartile 3, and all age groups in quartile 4 have increased OR (95% CI). For males, subjects aged ≤69 years in quartiles 2 and 3, and all age groups in quartile 4 had increased OR (95% CI) (Table 3).

### 3.5. The Likelihood of Having Serum Urate >420 *μ*mol/L with Serum Triglycerides Increasing

Subjects were classified into normal triglycerides and hypertriglyceridemia. After adjusting for confounders, the OR (95% CI) for suffering serum urate > 420 *μ*mol/L was 2.50 (2.20–2.84) in females with hypertriglyceridemia than those with normal triglycerides; while in males, it was 1.93 (1.87–2.00), which was much lower than that in females (Table 4). In age-stratified groups, the OR for having serum urate > 420 *μ*mol/L ranged from 1.90 to 3.69 in females and 1.83 to 2.07 in males, respectively. There was a trend of decrease in OR with aging in females but not in males (Table 4).

In the multivariable-adjusted logistic model, with each 1 mmol/L increment in triglycerides, the OR (95% CI) for having serum urate > 420 *μ*mol/L was 2.06 (1.89–2.24) in females, which was significantly higher than that in males (OR, 1.70, 95% CI, 1.66–1.74). In sex- and age-stratified subgroup analysis, the increased OR (95% CI) retained in each group. There was a trend of decrease in OR with aging in both females and males (Table 4).

## 4. Discussion

The major findings of our study were that serum urate progressively increased with serum triglycerides raising, and the likelihood for developing serum urate >420 *μ*mol/L significantly increased with elevated triglycerides. In addition, females had higher likelihood for developing serum urate >420 *μ*mol/L with triglycerides raising than males compared with their respective controls.

The prevalence of hypertriglyceridemia and hyperuricemia has gradually increased in recent decades. There is few evidence linking triglycerides as a risk factor for increased serum urate levels. Although a Mendelian randomization study provides a causal role of triglycerides in raising serum urate levels in males [14], the exact mechanisms remain elusive. It is well known that ATP is needed for fatty acid synthesis and triglycerides anabolism [18], while ATP depletion leads to the accumulation of AMP and overproduction of uric acid [8]. Thus, whether the ATP consumption during triglycerides synthesis in the liver could result in excess generation of uric acid needs to be clarified. Besides, whether triglycerides metabolism in the instestine could decrease the excretion of uric acid in the gut possesses another possible mechanism for the increased serum urate with elevated triglycerides. Uncovering these underlying mechanisms will undoubtedly clarify the causality between triglycerides and uric acid.

In our study, we found that with triglycerides increasing, the increased likelihood for developing serum urate >420 *μ*mol/L was higher in females than in males compared with their respective controls. Our previous study showed that as one gained weight, females had higher likelihood to develop hyperuricemia than males compared with their respective normal-weight controls [7]. Thus, it seems that though prevalence of hyperuricemia was much lower in females than in males [7, 19], women was more vulnerable to suffer hyperuricemia with elevated body mass index or triglycerides. Estrogen was demonstrated to have uricosuric effect and contribute to the lower levels of serum urate in females [20]. Nevertheless, on the other hand, whether estrogen could interact with body weight or triglycerides to intensify their urate-raising effect needs further investigation.

Our results showed that serum total cholesterol and LDL-C were in a positive correlation with serum urate. Nevertheless, the increment of serum urate was very small. In another study using data from the seventh Korea National Health and Nutrition Examination Survey 2016-2017, a similar positive trend between serum total cholesterol, LDL-C, and serum urate was found. And also, serum urate levels fluctuated in a small range with changes in serum total cholesterol and LDL-C after adjusting for multiple confounders [21]. Thus, total cholesterol and LDL-C alone may have little influence on serum urate changes, but may have synergistic effect on serum urate levels , sinceboth serum urate levels and the likelihood for developing hyperuricemia increased gradually with increasing numbers of dyslipidemia components [21].

For HDL-C, in females, there was an increase in serum urate as HDL-C increasing from 0.41  to 0.81 mmol/L. Nevertheless, since only a small number of subjects were within this range (*n* = 214), this trend of increase may not represent the real relationship between serum HDL-C and serum urate in females. Large number of subjects with HDL-C ≥ 0.41 and < 0.81 mmol/L are needed to uncover the real relationship between serum HDL-C and serum urate in this range of HDL-C.

Accumulating evidence supports the common risk factors for both hyperuricemia and dyslipidemia, such as high fructose intake, sedentary lifestyle, obesity, and genetic factors [22–24]. Some lipid-modifying agents such as atorvastatin, simvastatin, and fenofibrate could decrease serum urate levels independent of their lipid-lowering effect [25, 26]. The existence of genetic pleiotropy adds further evidence for the existence of common upstream pathological factors influencing both uric acid and lipid profiles [24]. Hence, measures to control dyslipidemia and lower serum urate at the same time were needed; these measures include lifestyle changes, exercise, and medications. Furthermore, the detection and treatment of comorbidities associated with dyslipidemia and hyperuricemia, such as obesity, hypertension, and dysglycemia, should also be taken into consideration in the clinical setting.

The strengths of the current study include the large sample size, providing enough power to study the association between lipid profiles and serum urate and to analyze the association in sex- and age-stratified groups. In addition, data collection was based on medical records rather than self-reported. However, there were also limitations in our study. First, causality could not be concluded because of the cross-sectional design. Second, since all the subjects were from the Health Examination Centre. A status of diseases and a history of medication use which may influence the relationship between lipid profiles and serum urate were not known. Nevertheless, in another study investigating the association between lipid profiles and serum urate, after adjusting for hypertension, diabetes, smoking status, alcohol intake, regular exercise, the administration of dyslipidemia medication, age, sex, waist circumference, body mass index, hemoglobin, eGFR, and urea nitrogen, serum total cholesterol, LDL-C, and triglycerides maintained their positive association with serum urate (negative for HDL-C) [21]. Thus, it seems that comorbidities and dyslipidemia medication use may not significantly change the association between lipid profiles and serum urate.

In conclusion, serum urate progressively increased with triglycerides increasing. The likelihood for suffering serum urate >420 *μ*mol/L increased with triglycerides raising. Females were in a higher likelihood for developing serum urate >420 *μ*mol/L with elevated triglycerides than males compared with their respective controls. Serum urate fluctuated in a small range with changes in total cholesterol, LDL-C, or HDL-C. Further studies should be taken to uncover the underlying mechanism and causality between lipid profiles and serum urate.

## Figures and Tables

**Table 1:** Characteristics of the subjects.

	Total	Age (years)						*p* value
		18–29	30–39	40–49	50–59	60–69	70–99	
*Females*								
*N*	55,742	6,366	12,407	18,306	11,172	5,334	2,157	
Age (years)	44 (36–52)	27 (25–28)	35 (32–37)	44 (42–47)	53 (51–56)	63 (61–66)	74 (71–77)	<0.001
Body mass index (kg/m^2^)	22.57 ± 3.00	20.51 ± 2.51	21.67 ± 2.70	22.76 ± 2.77	23.53 ± 2.93	23.98 ± 3.06	23.74 ± 3.21	<0.001
Serum urate (*μ*mol/L)	281.61 ± 58.44	283.35 ± 54.51	275.42 ± 55.89	272.48 ± 55.77	290.21 ± 59.47	296.24 ± 61.99	308.72 ± 68.73	<0.001
LDL-C (mmol/L)	2.78 ± 0.74	2.39 ± 0.63	2.53 ± 0.65	2.78 ± 0.70	3.08 ± 0.74	3.15 ± 0.76	2.99 ± 0.81	<0.001
HDL-C (mmol/L)	1.51 ± 0.30	1.53 ± 0.29	1.50 ± 0.30	1.51 ± 0.30	1.51 ± 0.31	1.50 ± 0.32	1.53 ± 0.33	<0.001
Total cholesterol (mmol/L)	4.78 ± 0.86	4.30 ± 0.70	4.44 ± 0.73	4.76 ± 0.79	5.18 ± 0.83	5.29 ± 0.85	5.13 ± 0.90	<0.001
Triglycerides (mmol/L)	1.09 (0.80–1.53)	0.82 (0.65–1.10)	0.92 (0.71–1.26)	1.09 (0.82–1.48)	1.32 (0.98–1.81)	1.42 (1.05–1.91)	1.42 (1.07–1.85)	<0.001
FPG (mmol/L)	4.93 ± 0.51	4.68 ± 0.43	4.82 ± 0.45	4.91 ± 0.48	5.07 ± 0.53	5.19 ± 0.54	5.21 ± 0.58	<0.001
Serum creatinine (*μ*mol/L)	55.00 (49.80–60.70)	53.50 (48.80–58.80)	53.80 (48.80–59.00)	54.70 (49.50–60.30)	56.20 (50.80–62.00)	57.00 (51.40–63.40)	59.80 (53.70–65.90)	<0.001
eGFR (mL/min/1.73 m^2^)	108.47 ± 13.31	124.76 ± 7.96	117.65 ± 8.18	108.74 ± 8.50	100.58 ± 8.69	93.00 ± 8.35	84.32 ± 8.09	<0.001

*Males*								
*N*	66,609	5,942	14,258	21,385	13,978	7,253	3,793	
Age (years)	45 (37–54)	27 (25–28)	35 (32–37)	44 (42–47)	53 (51–56)	63 (61–66)	75 (72–79)	<0.001
Body mass index (kg/m^2^)	24.31 ± 2.95	23.14 ± 3.21	24.19 ± 3.04	24.64 ± 2.85	24.64 ± 2.74	24.27 ± 2.86	23.53 ± 2.95	<0.001
Serum urate (*μ*mol/L)	385.79 ± 71.80	398.85 ± 71.86	395.95 ± 70.82	387.31 ± 70.71	381.23 ± 71.47	368.43 ± 71.69	368.64 ± 71.66	<0.001
LDL-C (mmol/L)	3.02 ± 0.74	2.78 ± 0.72	2.97 ± 0.72	3.09 ± 0.73	3.10 ± 0.75	3.04 ± 0.76	2.86 ± 0.79	<0.001
HDL-C (mmol/L)	1.29 ± 0.29	1.27 ± 0.27	1.26 ± 0.28	1.27 ± 0.28	1.29 ± 0.29	1.36 ± 0.31	1.40 ± 0.32	<0.001
Total cholesterol (mmol/L)	4.85 ± 0.83	4.49 ± 0.79	4.76 ± 0.80	4.93 ± 0.81	4.96 ± 0.83	4.92 ± 0.85	4.76 ± 0.89	<0.001
Triglycerides (mmol/L)	1.49(1.07–2.08)	1.24(0.90–1.78)	1.52(1.08–2.14)	1.61(1.16–2.22)	1.56(1.13–2.14)	1.34(0.98–1.84)	1.21(0.89–1.63)	<0.001
FPG (mmol/L)	4.97 ± 0.56	4.76 ± 0.48	4.85 ± 0.51	4.95 ± 0.55	5.06 ± 0.58	5.16 ± 0.59	5.22 ± 0.59	<0.001
Serum creatinine (*μ*mol/L)	77.00 (70.20–84.20)	77.20 (71.00–83.60)	77.30 (70.70–84.00)	76.90 (70.20–84.10)	76.80 (69.90–84.30)	76.60 (69.40–84.50)	78.20 (71.00–85.20)	<0.001
eGFR (mL/min/1.73 m^2^)	101.48 ± 13.13	116.30 ± 10.33	109.76 ± 10.24	102.70 ± 9.94	96.15 ± 9.63	89.88 ± 8.99	82.03 ± 7.79	<0.001

FPG: fasting plasma glucose; LDL-C: low-density lipoprotein cholesterol; HDL-C: high-density lipoprotein cholesterol; eGFR: estimated glomerular filtration rate.

**Table 2:** Sex-and age-stratified differences (95% confidence intervals) in serum urate levels (*μ*mol/L) according to serum triglycerides quartiles.

	Total	Age (years)					
		<29	30–39	40–49	50–59	60–69	70–99
*Females*							
Q1	Reference	Reference	Reference	Reference	Reference	Reference	Reference
*Q2*							
Crude	8.91 (7.57–10.25)	4.9 (1.11–8.68)	6.7 (3.97–9.43)	9.66 (7.44–11.88)	11.53 (8.48–14.58)	14.18 (9.59–18.78)	16.91 (8.99–24.84)
Adjusted^a^	6.85 (5.57–8.13)	2.4 (−1.22–6.01)	4.97 (2.39–7.55)	6.93 (4.81–9.06)	8.98 (6.07–11.89)	10.41 (6.06–14.75)	13.14 (5.76–20.53)
*Q3*							
Crude	19.36 (18.02–20.69)	8.6 (4.92–12.29)	16.23 (13.52–18.94)	17.01 (14.79–19.23)	20.8 (17.75–23.85)	23.52 (18.92–28.12)	32.46 (24.48–40.44)
Adjusted^a^	14.15 (12.84–15.46)	5.32 (1.79–8.86)	12.48 (9.9–15.06)	12.17 (10.03–14.31)	15.98 (13.05–18.91)	16.26 (11.87–20.66)	23.39 (15.84–30.93)
*Q4*							
Crude	39.74 (38.40–41.08)	25.45 (21.73–29.18)	33.97 (31.25–36.69)	36.91 (34.7–39.12)	37.61 (34.57–40.66)	39.68 (35.07–44.3)	50.2 (42.24–58.16)
Adjusted^a^	30.46 (29.08–31.85)	17.84 (14.16–21.52)	25.78 (23.11–28.45)	29.29 (27.09–31.48)	30.95 (27.98–33.92)	30.81 (26.36–35.27)	38.85 (31.26–46.43)

*Males*							
Q1	Reference	Reference	Reference	Reference	Reference	Reference	Reference
*Q2*							
Crude	18.19 (16.70–19.67)	15.44 (10.44–20.44)	19.25 (16.10–22.40)	22.52 (19.93–25.11)	17.19 (14.55–21.03)	18.27 (13.76–22.79)	15.26 (8.96–21.56)
Adjusted^a^	11.58 (10.16–13.00)	9.13 (4.35–13.91)	12.74 (9.69–15.78)	15.68 (13.18–18.19)	10.95 (7.85–14.05)	10.79 (6.48–15.10)	8.57 (2.52–14.62)
*Q3*							
Crude	34.35 (32.87–35.84)	30.34 (25.40–35.27)	37.15 (34.00–40.30)	37.16 (34.58–39.75)	33.54 (30.28–36.81)	30.93 (26.44–35.42)	23.15 (16.81–29.49)
Adjusted^a^	23.57 (22.11–25.02)	18.74 (13.89–23.59)	24.98 (21.85–28.10)	27.19 (24.65–29.73)	23.00 (19.84–26.17)	19.82 (15.47–24.17)	11.52 (5.32–17.71)
*Q4*							
Crude	52.30 (50.81–53.78)	54.67 (49.72–59.62)	55.44 (52.29–58.59)	52.23 (49.65–54.82)	49.74 (46.48–52.99)	54.93 (50.43–59.44)	44.63 (38.32–50.95)
Adjusted^a^	37.23 (35.74–38.72)	34.96 (29.83–40.09)	39.32 (36.09–42.54)	39.42 (36.83–42.00)	35.50 (32.29–38.71)	39.06 (34.59–43.54)	30.21 (23.9–36.53)

^a^Adjusting for age, body mass index, fasting plasma glucose, and estimated glomerular filtration rate.

**Table 3:** The OR (95% confidence interval) of serum urate > 420 *μ*mol/L according to serum triglycerides quartiles.

	Total	Age (years)					
		<29	30–39	40–49	50–59	60–69	70–99
*Females*							
Q1	Reference	Reference	Reference	Reference	Reference	Reference	Reference
*Q2*							
Crude	1.54 (1.19–2.02)	1.99 (0.86–4.95)	1.93 (1.09–3.54)	1.37 (0.76–2.52)	1.23 (0.78–1.95)	2.18 (1.21–4.14)	1.56 (0.84–3.01)
Adjusted^a^	1.29 (0.99–1.70)	1.75 (0.76–4.38)	1.69 (0.95–3.12)	1.09 (0.60–2.00)	1.09 (0.69–1.73)	1.93 (1.06–3.68)	1.40 (0.74–2.72)
*Q3*							
Crude	2.92 (2.30–3.72)	2.15 (0.96–5.25)	2.39 (1.38–4.31)	2.66 (1.59–4.62)	2.14 (1.44–3.27)	3.27 (1.87–6.06)	2.55 (1.43–4.73)
Adjusted^a^	2.02 (1.59–2.61)	1.78 (0.79–4.38)	1.84 (1.05–3.35)	1.84 (1.09–3.22)	1.72 (1.14–2.63)	2.48 (1.40–4.63)	2.06 (1.14–3.88)
*Q4*							
Crude	6.96 (5.60–8.75)	6.89 (3.47–15.72)	6.05 (3.72–10.50)	6.81 (4.31–11.39)	4.28 (2.97–6.33)	5.57 (3.29–10.1)	4.08 (2.38–7.41)
Adjusted^a^	4.02 (3.18–5.13)	4.22 (2.05–9.83)	3.64 (2.19–6.40)	4.06 (2.52–6.90)	3.33 (2.29–4.98)	4.11 (2.40–7.53)	3.08 (1.77–5.67)

*Males*							
Q1	Reference	Reference	Reference	Reference	Reference	Reference	Reference
*Q2*							
Crude	1.62 (1.54–1.71)	1.42 (1.20–1.68)	1.74 (1.56–1.95)	1.79 (1.63–1.97)	1.54 (1.37–1.73)	1.84 (1.53–2.22)	1.30 (1.02–1.65)
Adjusted^a^	1.41 (1.34–1.49)	1.22 (1.02–1.45)	1.51 (1.35–1.70)	1.55 (1.40–1.70)	1.33 (1.18–1.50)	1.56 (1.29–1.90)	1.09 (0.85–1.40)
*Q3*							
Crude	2.43 (2.31–2.56)	2.13 (1.82–2.50)	2.68 (2.41–2.99)	2.68 (2.44–2.93)	2.26 (2.02–2.54)	2.58 (2.16–3.1)	1.64 (1.30–2.07)
Adjusted^a^	1.96 (1.86–2.07)	1.63 (1.38–1.93)	2.06 (1.85–2.31)	2.19 (1.99–2.41)	1.83 (1.62–2.06)	2.05 (1.70–2.47)	1.21 (0.95–1.55)
*Q4*							
Crude	3.80 (3.62–4.00)	4.09 (3.49–4.80)	4.32 (3.89–4.80)	3.84 (3.52–4.20)	3.46 (3.09–3.87)	4.40 (3.70–5.25)	2.80 (2.25–3.50)
Adjusted^a^	2.84 (2.69–3.00)	2.66 (2.23–3.17)	3.10 (2.76–3.47)	2.99 (2.72–3.28)	2.61 (2.32–2.94)	3.19 (2.66–3.84)	2.01 (1.59–2.55)

^a^Adjusting for age, body mass index, fasting plasma glucose, and estimated glomerular filtration rate.

**Table 4:** The OR (95% confidence interval) of serum urate > 420 *μ*mol/L according to normal triglycerides and hypertriglyceridemia.

	Total	Age (years)					
		<29	30–39	40–49	50–59	60–69	70–99
*Females*							
Normal triglycerides	Reference	Reference	Reference	Reference	Reference	Reference	Reference
*Hypertriglyceridemia*							
Crude	3.71 (3.29–4.18)	6.21 (3.84–9.74)	3.72 (2.69–5.07)	4.00 (3.05–5.22)	2.79 (2.22–3.53)	2.76 (2.04–3.74)	2.31 (1.64–3.25)
Adjusted^a^	2.50 (2.20–2.84)	3.69 (2.17–6.09)	2.25 (1.59–3.13)	2.87 (2.16–3.80)	2.41 (1.90–3.07)	2.33 (1.71–3.18)	1.90 (1.34–2.71)

*1* *mmol/L increment*							
Crude	2.60 (2.41–2.79)	3.71 (2.77–4.91)	2.67 (2.20–3.21)	3.06 (2.59–3.59)	2.10 (1.80–2.43)	2.18 (1.79–2.64)	1.81 (1.42–2.29)
Adjusted^a^	2.06 (1.89–2.24)	2.62 (1.87–3.61)	1.98 (1.59–2.45)	2.50 (2.08–2.98)	1.89 (1.61–2.21)	1.96 (1.59–2.41)	1.56 (1.20–2.01)

*Males*							
Normal triglycerides	Reference	Reference	Reference	Reference	Reference	Reference	Reference
*Hypertriglyceridemia*							
Crude	2.35 (2.27–2.43)	2.69 (2.40–3.03)	2.46 (2.30–2.64)	2.30 (2.17–2.44)	2.19 (2.04–2.36)	2.51 (2.24–2.81)	2.21 (1.87–2.61)
Adjusted^a^	1.93 (1.87–2.00)	1.97 (1.73–2.24)	1.96 (1.82–2.11)	1.96 (1.84–2.09)	1.83 (1.69–1.98)	2.07 (1.83–2.33)	1.84 (1.54–2.20)

*1* *mmol/L increment*							
Crude	1.94 (1.90–1.98)	2.24 (2.06–2.43)	2.02 (1.93–2.12)	1.92 (1.84–1.99)	1.86 (1.77–1.95)	2.04 (1.88–2.21)	1.82 (1.62–2.05)
Adjusted^a^	1.70 (1.66–1.74)	1.80 (1.64–1.97)	1.73 (1.65–1.83)	1.72 (1.65–1.79)	1.64 (1.56–1.73)	1.76 (1.61–1.91)	1.56 (1.37–1.77)

^a^Adjusting for age, body mass index, fasting plasma glucose, and estimated glomerular filtration rate.

## Data Availability

Access to data is restricted since the government' requests.
